# Zebrafish for Personalized Regenerative Medicine; A More Predictive Humanized Model of Endocrine Disease

**DOI:** 10.3389/fendo.2020.00396

**Published:** 2020-07-17

**Authors:** Babak Arjmand, Akram Tayanloo-Beik, Najmeh Foroughi Heravani, Setareh Alaei, Moloud Payab, Sepideh Alavi-Moghadam, Parisa Goodarzi, Mahdi Gholami, Bagher Larijani

**Affiliations:** ^1^Cell Therapy and Regenerative Medicine Research Center, Endocrinology and Metabolism Molecular-Cellular Sciences Institute, Tehran University of Medical Sciences, Tehran, Iran; ^2^Metabolomics and Genomics Research Center, Endocrinology and Metabolism Molecular-Cellular Sciences Institute, Tehran University of Medical Sciences, Tehran, Iran; ^3^Obesity and Eating Habits Research Center, Endocrinology and Metabolism Molecular-Cellular Sciences Institute, Tehran University of Medical Sciences, Tehran, Iran; ^4^Brain and Spinal Cord Injury Research Center, Neuroscience Institute, Tehran University of Medical Sciences, Tehran, Iran; ^5^Department of Toxicology and Pharmacology, Toxicology and Poisoning Research Center, Faculty of Pharmacy, Tehran University of Medical Sciences, Tehran, Iran; ^6^Endocrinology and Metabolism Research Center, Endocrinology and Metabolism Clinical Sciences Institute, Tehran University of Medical Sciences, Tehran, Iran

**Keywords:** animal models, endocrine disease, personalized medicine, regeneration, zebrafish

## Abstract

Regenerative medicine is a multidisciplinary field that aims to determine different factors and develop various methods to regenerate impaired tissues, organs, and cells in the disease and impairment conditions. When treatment procedures are specified according to the individual's information, the leading role of personalized regenerative medicine will be revealed in developing more effective therapies. In this concept, endocrine disorders can be considered as potential candidates for regenerative medicine application. Diabetes mellitus as a worldwide prevalent endocrine disease causes different damages such as blood vessel damages, pancreatic damages, and impaired wound healing. Therefore, a global effort has been devoted to diabetes mellitus investigations. Hereupon, the preclinical study is a fundamental step. Up to now, several species of animals have been modeled to identify the mechanism of multiple diseases. However, more recent researches have been demonstrated that animal models with the ability of tissue regeneration are more suitable choices for regenerative medicine studies in endocrine disorders, typically diabetes mellitus. Accordingly, zebrafish has been introduced as a model that possesses the capacity to regenerate different organs and tissues. Especially, fine regeneration in zebrafish has been broadly investigated in the regenerative medicine field. In addition, zebrafish is a suitable model for studying a variety of different situations. For instance, it has been used for developmental studies because of the special characteristics of its larva. In this review, we discuss the features of zebrafish that make it a desirable animal model, the advantages of zebrafish and recent research that shows zebrafish is a promising animal model for personalized regenerative diseases. Ultimately, we conclude that as a newly introduced model, zebrafish can have a leading role in regeneration studies of endocrine diseases and provide a good perception of underlying mechanisms.

## Introduction

Through the past few decades, the incidence of different diseases presents an alarming increase rate and in most cases, it is due to the increasing sedentary lifestyle in industrialized countries. As the most prevalent disease, cardiovascular diseases are known as the major causes of morbidity and mortality in many countries all over the world. However, other types of disorders including endocrine and metabolic ones consist of various types of diseases, which with their remarkably increasing rate, affect the patients' quality of life. Today, diabetes mellitus (DM), obesity, and fatty liver disease are considered as leading concerns in public health (1, 2). Hence, an international rising effort is made to effectively cure these disorders or, at least, diminish their different side effects. Accordingly, any novel and effective therapeutic approaches will be valuable. In this concept, through recent decades, cell therapy, and regenerative medicine hold a great promise in representing novel therapies for several types of diseases. Additionally, personalized medicine as a novel field plays an exceptional role in the optimization of new therapies based on the specific genome characteristics of individuals (3). In spite of considerable progress in the personalized regenerative medicine field, there are still several uncertainties that need to be determined. For addressing these uncertainties, multiple investigations are necessary to establish more applicable protocols for clinical applications. Since there are serious prohibitions in performing tests and researches on human, as alternatives, animal models providing the possibility of various investigations to discover the mechanism of different diseases (4–6). Accordingly, a variety of animal species are used to simulate human disease conditions. Animal models could mimic human pathophysiological conditions. Their importance and priority are determined according to their level of similarity to human physio-anatomical characteristics. In the second degree, availability, easy handling, cost efficiency, fecundity, size, and ethical aspect are other determining factors. For instance, in the field of endocrinology, different animal species have been modeled in obesity, diabetes, metabolic syndrome, and etc. but most frequently used are mouse models (7, 8). However, in the matter of regeneration studies, scientists need a model with something more than mentioned characteristics, a model that possesses regeneration capacity. Hereupon, zebrafish, a vertebrate model, presents specific properties that make it a suitable choice for investigating in different fields of biomedicine. Unlike several animal models, zebrafish has regeneration capability in its organs such as fins, central nervous system (CNS), heart, pancreas, liver, and kidney. Regarding this given capacity, it is used for different models of injury for example in cardiovascular, neurological, and metabolic diseases (9, 10). Additionally, zebrafish is used as an animal model in personalized medicine for studying different diseases like cancers as avatars due to its beneficial advantages which are discussed in this review (11, 12). Moreover, it is mentioned that zebrafish was used in investigating one of the unique features in humans, the gut microbiota, in order to find the associations between metabolic disorders and intestinal microbiota (13). Ultimately, the present review is specially focused on the application of the zebrafish model for studying endocrine disease from the personalized regeneration medicine view of point.

## Personalized Regenerative Medicine

Empowered by the knowledge and techniques of several different disciplines, regenerative medicine is an emerging field of research and clinical application that can be defined as any procedure aiming to restore the function of lost tissue (due to damage caused by trauma, disease, aging, or congenital defects) through regenerating or replacing cells by means of stem cell therapy, molecular activators, tissue engineering, etc. Although there are few exceptions like liver, human body's own, repair mechanism replaces the lost cells with scar tissue resulting in function loss. Stem cell therapy is the main part of regenerative therapies, which uses embryonic, induced pluripotent or autologous stem cells to replace the damaged tissue and gain function, thereby potentially reducing chronic disease burden and lifelong requirement of medication (3). Personalized medicine is a novel field that provides the potential to choose the optimal therapy for individuals based on data such as genomic profile and pharmacogenomics. Personalized medicine can significantly increase patient compliance with the drug and subsequently ensure better patient care while reducing costs (3). Current treatment strategies in facing chronic diseases are limited to preventive and alleviative measures that are of limited value to patients. Personalized regenerative medicine lays the ground for developing effective therapies for these conditions. There are promising observations in using both stem cell and pharmacological approaches to treat musculoskeletal conditions like age-related muscle loss and sarcopenia in preclinical studies (14, 15). There are numerous examples of successful stem cell clinical application in regenerative medicine (Table 1), the most known include: bone marrow transplantation to treat leukemia and using stem cells to heal burnt skin (32, 33). The most common registered trials in the field of regenerative medicine are focused on cardiovascular diseases, nervous system diseases and musculoskeletal conditions (34). There are reports of successful limbal stem cell use to treat corneal damage. In treating type 1 diabetes mellitus (T1DM), intravenous administration of CD34+ bone marrow hematopoietic stem cells have shown the most promising results in restoring pancreatic function. Stem cell therapy has also been studied in treating cancers, obesity, Parkinson's disease, hearing recovery, Alzheimer's disease, etc. (6, 35–39). Cardiovascular diseases are a major area of concern as they have a high incident, morbidity and mortality rate and include a variety of chronic conditions such as myocardial infarction (MI), atherosclerosis and aneurysms. In order to further study these conditions porcine animal models have been introduced recently. These models fit the criteria needed to study both stem cell and pharmacologic treatment options for cardiovascular diseases (40). Animal models fill the gap between *in vitro* studies and human clinical application of any new treatment. They help assess the risks and efficacy of the newly introduced method prior to human clinical trials, they also increase the chance of method's success in human application (41).

**Table 1:** The most prevalent fields and studies in personalized regenerative medicine.

**Field**	**Objective**	**Study**
Cardiovascular disorders	Generating atrial and ventricular cells from hPSCs	Stem cells were induced with 10B/6A, Activin-A and BMP-4 to generate ventricular cells. Stem cells were induced with Retinol and Retinoic acid to differentiate into atrial cells (16).
	Drug discovery for long QT syndrome	iPCS derived from skin cells with Timothy syndrome were used to model Long QT syndrome to assess the effects of different drugs (17).
	Restoring contractile capacity of injured heart	Electrical integration of hESCs-derived cardiomyocytes to the heart to restore the contractile activity of heart while reducing arrhythmias (18).
	Repairing the scar tissue in heart using stem cells	Intracoronary administration of autologous cardiosphere-derived cells decreased scar size, increased viable myocardium, and improved regional function of infarcted myocardium at 1 year post-treatment (19).
	Repairing the heart after myocardial infarction using non-stem cell therapy	The sequential delivery of IGF-1 and HGF from an injectable alginate biomaterial attenuated infarct expansion, preserved scar thickness, and reduced scar fibrosis while increasing angiogenesis and mature blood vessel formation at the infarct tissue (20).
		FGF1/p38 MAP kinase inhibitor administration after acute myocardial injury reduces the scar tissue and improves function in rats (21).
Nervous System Disorders	Study Parkinson's disease	hiPCS in primate models form dopaminergic neurons improved Parkinson's symptoms (22).
	Study age related macular degeneration	An Individual's own iPCS were used to generate retinal pigmented epithelium (23).
	Neural precursor cell transplantation effects on myelination to treat Multiple Sclerosis	Neural precursor cell transplantation increases host myelin regeneration caused by chronic demyelination (24).
	Study Alzheimer's disease	Genetically or pharmacologically upregulating growth factors like brain-derived neurotrophic factor, IGF-1, nerve growth factor, and vascular endothelial growth factor that induce neurogenesis (25).
		ESCs, MSCs, brain-derived neural stem cells, and PSCs were transplanted in rodent models to improve cognitive ability and alleviate Alzheimer's symptoms.
		UCB-MSCs are the common stem cells in human trials in Alzheimer's. Ischaemia tolerant allogeneic human bone marrow derived MSCs are stem cells that are more compatible with the CNS physiological conditions produce more growth factors and are also being used in human trials.
Musculoskeletal conditions	Bone, cartilage, and tendon regeneration	Mostly MSC along with ESCs and iPSCs are used to treat cartilage, bone, and tendon repair. BMP2 and VEGF enriched scaffolds improve osteogenesis and vascularization (26).
	Cartilage repair	SDSCs transplantation in synovial joints show promising chondrogenic results (27).
Endocrine disorders	Possible treatments for diabetes	hPSCs and iPSCs have shown results in producing functional pancreatic lineage and β-like cells (28). Fibroblast, α-cell, hepatocyte, gastrointestinal tissue cell, and pancreatic exocrine cell reprogramming to β-cells (29).
	Possible treatments for obesity	BAT transplantation has had therapeutic results in rodent obesity models (30).
		FGF-21 reduced mean body weight of diet induced obese rats by 20% (31).

*Stem cells (typically MSCs) are the primary therapeutic choice in chronic disease research. Rats are the common choice as animal models*.

*BMP, Bone morphogenetic protein; ESCs, Embryonic stem cells; IGF, Insulin-like growth factor; HGF, Hepatocyte growth factor; FGF, Fibroblast growth factor; MAP, Mitogen-activated protein; CNS, Central Nervous system; VEGF, Vascular endothelial growth factor; iPCS, induced pluripotent stem cell; MSC, Mesenchymal Stem cell; hPSCs, Human pluripotent stem cells; UCB-MSCs, Umbilical cord blood-derived mesenchymal Stem cells; BAT, Brown adipose tissue; SDSCs, Synovium-derived stem cells*.

## Animal Models in Biomedicine Studies

Generally, a model in science is a simplified situation of a complexity that presents many common characteristics with the main version. In the animal view of point, a model is a particular animal that shares several pathophysiological similarities with human. In this case, the more suitable model is one that is phylogenetically closest to human species. Since tests and researches in humans have been banned and can raise a lot of ethical problems, animals are ideal alternatives that provide a possibility for scientists to do different studies (6). Animal modeling leads to extend science boundaries and astonishing improvements in clinical findings. Different results of medications, treatments, and cures can be investigated in animal models without any risk for human. Notably, choosing a suitable animal model is considered as a pivotal step in a preclinical study. In this context, selecting animals with the closest possible biological features to the target population in, considering that what is going to be tested in choosing animals, pointing the cost efficiency as a priority, conducting a proper literature review and a complete examination of similar studies, choosing the animal according to our needs, resources and possible help from different organizations all together are instructions which can provide better outcomes in animal studies (42, 43). Up to now, different species of animals have been modeled for various human disease conditions. From simple microorganisms to highly developed vertebrates are ranged in this category. According to the specific purpose of research, each of these animal models is considered as the gold standard in a particular situation. For instance, in order to study the complex system of the brain, vertebrates like Rat, Mouse, Zebrafish, Primates, with a highly developed nervous system and human resemblance are better choices. Additionally, different cellular and molecular studies and biochemistry mechanisms of the body are possible using small organisms such as sea snail, *Caenorhabditis elegans* (*C. elegans*), nematode, Drosophila (44). Among the mentioned animal models, mouse models are mammals broadly used in biomedical researches which can be caused astonishing progress in this field. Although they present undebatable numerous advantages, there are limitations to these models. Stress in mice is a notable factor that can lead to different confounds in the behavioral human studies. The need for large space for breeding, complexity in handling, relatively high cost of maintenance, and etc. are some other difficulties (45, 46). Accordingly, scientists have been introduced some alternatives to ease different processes in animal studies. Nowadays, Zebrafish (*Danio rerio*) as an emerging model draws attention to its unexceptional characteristics. Its small size, low cost of use, short generation time, need for small space, rapid reproduction, wide progeny, high homology with the human genome and physiology, and easy manipulation make it a suitable choice for different fields of biomedicine (47, 48).

## Zebrafish as a Powerful Translational Model

Zebrafish is a powerful vertebrate translational model for studying genome and mutations, drug discovery, toxicity, human neurological, endocrine, and cardiovascular disorders. There are specific traits of zebrafish that make it a suitable model for studies (49):

- Low cost maintenance

Due to its simple natural habitat and size, zebrafish can be housed efficiently with up to 40 fish in a tank with the size of a standard mouse cage. Additionally, in drug discovery studies expensive agents are used in smaller amounts resulting in a decrease in the overall budget.

- Size

Zebrafish are 2–3 cm in length which makes whole organ analysis or transplantation much easier. Although its size is an advantage in many procedures it can mean inadequate sample for a number of analysis.

- Fecundity

Zebrafish have a large clutch size of 200–300 per fish, which makes parallel hypotheses testing and rare genetic incident studies rather easier. These fish also have a rapid maturation time which helps with reducing total research duration.

- Larvae and embryo optical clarity

This is a unique feature in zebrafish that allows genetic studies using non-invasive imaging techniques with labeled genes to be conducted easily on this model.

- External fertilization and our understanding of its genome

The information we have on zebrafish genome and its external fertilization facilitates embryo manipulation and makes it a suitable model for forward and reverse genetics, positional cloning and genome editing using zinc finger nuclease, clustered regularly interspaced short palindromic repeats(CRISPR), Transcription activator-like effector nuclease(TALEN) and transgenesis.

- Ethical aspect

Using fish raises far fewer ethical obstacles than using a mammalian model. Any animal model to study human diseases has some limitations which in this case are:

Fundamental differences of systems like reproductive and respiratory systems between zebrafish and humans.

- Aquatic habitat

The nature of its habitat is also a point of strength, but it is also a challenge for some procedures such as electrocardiography (EKG) (50).

### Zebrafish as a Model for Neurological Disorders

Zebrafish can also be used as a valid neurological model since it has the same neural structures as humans in the brain specifically at the molecular level. It produces neurotransmitters such as serotonin, gamma-amino butyric acid (GABA), dopamine, histamine, etc. Zebrafish neural cells are also similar to human's microglia, astrocytes, motor neurons, etc. Hence, it is used for studying attention deficit hyperactivity disorder (ADHD) and other neurological disorders. The genome of the zebrafish has similar genes to those mutated in familial Alzheimer's disease and it has been used in Alzheimer's studies (51–53).

### Zebrafish as a Model for Cardiovascular Diseases

Zebrafish is also being used widely as an important animal model in cardiovascular studies due to its similarities with human cardiovascular system in features like heart rate and genes involved in generation and the ability to regenerate its heart (54–56). It has been vastly used in many researches, studying early heart development and angiogenesis molecular mechanism, cardiomyopathy and mechanical vessel injury (57, 58). Another reason for zebrafish's convenience as a cardiovascular model is the imaging methods and physiological assays that have been developed to better study zebrafish cardiovascular system (59). Researchers have developed atherosclerosis in zebrafish with high fat diet to study inflammatory vessel injury (60).

## Regeneration Capability of Zebrafish

Zebrafish is known as an informative animal model for studying mechanisms of regeneration. This fascinating feature is due to its high regenerative capability and amenability to various genetic approaches. This animal has a remarkable capacity of organ regeneration including fins, CNS, heart, pancreas, liver and kidney, some of which are introduced (61). Unlike adult mammalian cardiac muscle, zebrafish has the ability of heart regeneration, indicating a potential way to decrease the mortality and morbidity of cardiac diseases especially MI. Accordingly, there are different injury models being used to stimulate heart regeneration in zebrafish such as cryoinjury (mimicking aspects of MI) and genetic ablation (inducing end-stage heart failure and removing a large amount of cardiomyocytes). Based on these studies, there are two major components of heart regeneration: proliferation of existing cardiomyocytes and an environment for stimulating muscle generation. The cell proliferation increases sharply after tissue damage based on various underlying processes, for instance, different genes are expressed in the endocardium. The epicardium proliferates and releases diverse factors that facilitate the proliferation and it acts as a vascular support. Hence, there is now an evidence that the mature cardiomyocytes can be expanded and regenerate, based on the zebrafish researches (48, 61). Another surprising area in zebrafish is neural regeneration. Neuronal cell loss results in mental, motor or visual impairment in humans while the capacity of regenerating neurons (in the brain, spinal cord, and retina) is found in zebrafish. In order to direct retinal development, Muller glia are induced to yield multipotent neuronal progenitor cells which migrate to the damaged site and differentiate in to the target cell type. Somehow similarly, this process happens in zebrafish by up regulating expression of many molecules in different pathways and remarkable specificity. In the spinal cord, zebrafish can produce new axonal projections, neurons, and interneurons at the damaged region. The location of progenitor radial glial cell addresses the type of regenerated neurons which is dependent on various gene expressions in each site. The axonal regeneration is based on radial glial cells that infiltrate the lesion site and proliferate, which is affected by fibroblast growth factor (FGF) signaling. Finally, the regeneration of the brain has been reviewed exclusively and it was revealed that the radial glial cells in the ventricles proliferate making progenitor cells as a result of the surgical lesions of the telencephalon. This process, similar to previous ones, involves many signaling pathways like Notch signaling which is known as an inhibitory pathway in the retina and motor neuron regeneration. Also, inflammation seems to be another essential part (61). Metabolic diseases such as diabetes, non-alcoholic steatohepatosis (NASH), obesity, and liver failure are of great importance regarding their increasing prevalence all over the world (62). Pancreas, as a major regulatory organ, has a limited ability of regeneration and its response to injury is usually through inflammation and repair mechanisms which result in diabetes and insulin dependence. On the contrarily, pancreas in adult zebrafish can recover after chemical treatment or surgery (pancreatectomy) without the help of insulin therapy. Histological analysis revealed that dividing insulin positive β cells are seen in both islets and pancreatic ducts, indicating that they might be a source of β cell progenitors. In addition, Centro-acinar cells may differentiate or α cells convert into β cells. Hence, this ability provides a platform for *in vivo* examination of the regenerating β cells to discover the related signals and pathways for reestablishing βcell function and glucose homeostasis (63, 64). Likewise, liver can regenerate following hepatectomy, drug induced hepatotoxicity, and hepatocyte ablation in adult zebrafish. After resecting a single lobe, the original lobular structure recovers, unlike the hyperplasia in mammals, based on expression of different factors and signals [ubiquitin like with PHD and finger domain1(uhrf1), prostaglandin E2(PGE2), and wingless-related integration site (WNT)] which are similar to mechanisms underlying regeneration after drug induced liver injury. Additionally, a novel method for inducing liver injury is nitroreductase (NTR)- mediated hepatocyte ablation, exhibiting the differentiation of biliary epithelial cells (BECs) to mature hepatocytes (65). All in all, since humans are rather incapable of regenerating some tissues and organs such as cardiac muscle and the CNS, there are two main options to overcome this challenge: application of an exogenous tissue or cell source or stimulation of the endogenous cells. Accordingly, to study the methodology of activating the endogenous cells, zebrafish is an appropriate animal model. Moreover, it has contributed to metabolic diseases researches including pathologic conditions in pancreas and liver by making the identification of signals and regeneration mechanisms easier. Therefore, it can be said that the emerging field of zebrafish can be used for therapeutic approaches (61).

## Humanized Zebrafish Model for Personalized Regenerative Medicine

As was mentioned in previous sections, regeneration is about re-establishing the structure and form of the tissue, organ, or organism via different modes such as the application of adult stem cells (66). Also, it varies from whole-body to cellular regeneration. Additionally, there are common pathways which have regulatory roles in the regeneration responses of different species including Wnt and Notch pathways. Misregulation of these pathways can cause cancerous tissue in humans (66). In other words, it seems that evolution related to the downregulation of regeneration responses can have a preventive effect on developing cancers in humans. In fact, it can be due to increased complexity of tissues and metabolism in mammals because of the dependence of mammal regeneration responses on age and tissue changes. However, the exact mechanisms are not fully understood (66, 67). Hence, understanding the mechanisms of regeneration and endogenous tissue repair is of great importance in the prospects of regenerative medicine (67). Accordingly, zebrafish is considered as an informative animal model for regenerative medicine due to its beneficial features including highly regenerative capacity and amenability to genetic manipulation, which makes it a therapeutic model for organ development and diseases, despite its limitations such as gene duplications (61, 66, 68). Further, zebrafish has become more and more popular in modeling human diseases for understanding the effects of genetic mutations, environmental disturbances, and drug development (69). In addition, one of the most prevalent areas in which zebrafish is a model for the development of this field is personalized cancer medicine (personalized medicine for cancer). Personalized medicine tailors the use of drugs and treatment strategies based on specific characteristics of the disease, primarily the underlying genetic mutations, in order to reach the optimal result of medication and other treatment strategies. Generally, in order to achieve the most efficient treatment, an understanding of the underlying pathophysiology and drug efficacy, animal model is crucial. Indeed, animal models help us to build knowledge and develop personalized treatments, especially for chronic diseases and cancers. Herein, the use of Avatars in cancer clinical trials is a great example of how personalized medicine can maximize the efficacy of therapies. In this method, the patient's cancer cells are xenotransplanted into avatars for determining drug efficacy. Currently, “humanized mice” are specified as the gold standard of xenograft assays. They are immunocompromised small mammals, transplanted with human cells, tissues, and organs. Although there are definitive benefits in using these models, their application is accompanied by some drawbacks. For example, difficulty in the evaluation of engraftment in the early steps, the adverse effects of irradiation regimes, prolonged engraftment assessing procedures, special maintenance requirements for immunosuppressed animals, and restriction in the number of animals that can be screened which consequently raise the overall cost and duration. All of these, are limitations that justify substituting more qualified alternatives (11, 70). It has now become clear that Zebrafish has many advantages over mice. Embryo transparency that enables *in-vivo* imaging of engrafted cells, high permeability to small molecules of chemotherapy drugs, providing a rejection-free environment with high proliferation rate of xenotransplanted cancer cells, enabling multiple drug tests with low cost housing and faster maturation and reproduction rate, less ethical issues along with similar chemo-sensitive responses with mice are what makes zebrafish a better avatar for personalized medicine in cancer studies (11, 12). Additionally, Zebrafish has a relatively identical genetic profile to humans that makes it a perfect tool for genetic manipulation. Eighty-two percent of proteins that code human genes are found to have close relations with zebrafish genes that most of them are thought to be associated with human diseases. Transgenic zebrafish models cause prolonged human-derived stem cell survival and differentiation through the expression of human cytokines (69). Eventually, regarding the genetically tractable characteristics of zebrafish, it can be developed into a transgenic model as one of the strategies of its application in personalized medicine in different fields of human diseases (3, 63, 69). Since many kidney diseases are caused by mono/polygenic mutations, zebrafish is used to both reveal the underlying genetic mutation and pathophysiology and model for drug screening in the hopes of personalized medicine) (71–73). On the other hand, zebrafish is distinctly useful in personalized medicine when treatment options are abundant, etiologies are not well-established, or the abnormality is rare; like endocrine disorders, cancers, and Duchene muscular dystrophy (11, 74, 75). Regarding endocrine and metabolic diseases, zebrafish is reported to have great potential as a model for investigating thyroid diseases including thyroid cancer, thyroid hormones, and its receptors defects. Although there are some differences, the related molecular mechanisms and components of the thyroid axis in zebrafish is comparable to mammals. Therefore, by using different strategies like transgenic lines, this model is suitable for developing new therapeutic approaches (76). Moreover, it was found that long-term exposure to environmental chemicals has caused physiological alterations and various disorders, some of which seem to be associated with changes in the endocrine system. The chemical substances that interfere with the components of the endocrine system are called endocrine-disrupting components (EDCs). There are some tools for detecting EDCs with specific limitations. Recently, zebrafish -due to its conservation with higher vertebrates- was used as an experimental model for parallel assessment of estrogenic, androgenic, and thyroid-disruptors by application of specific gene biomarkers and a transcriptomic platform. Using zebrafish contributed to a better understanding of related mechanisms and detection of EDCs (77). Additionally, since the zebrafish organ systems are similar to humans, especially those related to metabolic control for regulating whole-body energy homeostasis, it has been used to model different diseases in the field of metabolic diseases including obesity, diabetes, Non-alcoholic fatty liver disease (NAFLD), NASH, atherosclerosis, and even liver cancer (62, 78). Fundamental zebrafish metabolic mechanisms seem to be significantly similar to humans (79). Ezetimibe and Simvastatin helped reduce cholesterol levels in zebrafish in one study which can mean cholesterol regulation pathways of humans and zebrafish closely resemble (80). Research suggests similarities between human and zebrafish in peripheral nervous system development (81). Studies have also revealed that sympathomimetic and parasympathomimetic agents produce similar results in both human and zebrafish (82). Obesity researchers can also use zebrafish as a qualified animal model since adipose tissue development is based on the same principals in both species, studies also suggest similar genetic factors (83). However, there have been drawbacks in some cases due to late appearance of adipocyte tissue in zebrafish or lack of response to knocking out an important gene in the leptin pathway (84).

Studying NAFLD in zebrafish has many advantages (85, 86):

1. The possibility of visualizing the liver by staining the lipids
2. The feasibility of liver dissection in order to conduct analysis
3. Using radiolabeled agents to follow different metabolic pathways.

Using zebrafish as a model for steatosis revealed some promising anti-inflammatory agents to be risk factors especially in diabetic and obese patients (87). Understanding metabolic homeostasis in development is a very powerful tool for introducing new models that have the most resemblance with humans.

Researchers studying glucoregulation and response to metabolic and developmental disruption in zebrafish revealed mammalian similitude in glucoregulatory process in embryos by inducing hypoglycemia with 3-Mercaptopropionic acid (3-MPA). Transcript levels of phosphoenolpyruvate-carboxykinase1 (PCK1), INSB and INSA mRNA levels also supported the hypothesis. Zebrafish and mammals seem to be similar in glucose dynamic throughout embryonic development and Pck1 regulation (79). These findings present zebrafish as a perfect model for studying DM in humans (24). As previously mentioned, Zebrafish pancreatic β cells have a regeneration potential in response to damage. The possibility to visualize all cells *in vivo* makes zebrafish a powerful model for discovering genetic cues responsible for β-cells regenerative potential. Furthermore, studying molecular signals of regeneration revealed adenosine signaling role in β-cells proliferation (88). Overall, based on all these facts about the beneficial versatility and utility of zebrafish as a model in metabolic diseases and regenerative medicine, in order to introduce it as a potential personalized regenerative medicine model for metabolic disorders, manipulation of the gut microbiota might be a good example. Human gut microbiota is known to be unique and diverse which has an important role in developing diseases, inflammation, and different clinical outcomes (89, 90). Also, it is associated with personalized medicine, and it is possible that using specific microbiota and microbiome helps make favorable outcomes in treatment strategies (90). Additionally, changes in intestinal microbiota which include overproduction and release of bacterial endotoxins [lipopolysaccharides (LPS)] and increased small intestine permeability can cause liver damage which was studied in zebrafish. Inducing insulin resistance and liver inflammation may be involved in the development of NAFLD and changes in gut bacterial composition lead to obesity. Using prebiotic or probiotic drugs modifies the microbiota and changes the diet and pharmacological treatments of obesity (13, 91). Hereupon, in order to achieve a better understanding of associations between gut microbiota and obesity and its related disorders, zebrafish was used as an animal model since its composition of the microbiota is similar to mammals, in addition to other beneficial features of this animal (13). Germ-free zebrafish and adult zebrafish fed with different amounts of lipid are some examples of models studied and resulted in new possibilities for treating obesity and its associated metabolic disorders by manipulation of intestinal microbiota (13, 92). Concerning the role of gut microbiota in personalized medicine and pathogenesis of metabolic disorders such as obesity and NAFLD, zebrafish model could be a useful tool for realizing mechanisms of developing such disorders in the context of alteration of intestinal microbiota and developing possible therapeutic alternatives in personalized regenerative medicine (13, 90). Taken together, it can be concluded that unique features of zebrafish like high regenerative capacity, easier disease induction, low-cost housing, high maturation rate, and fecundity along with the utility of zebrafish in modeling endocrine and metabolic disorders, make it a better choice for developing personalized regenerative treatment. Treatments developed with zebrafish modeling are available faster and to a larger group of patients since this method produces faster results with lower cost (11, 63, 66).

## Zebrafish Models for Endocrine and Metabolic Diseases

**Figure 1:**
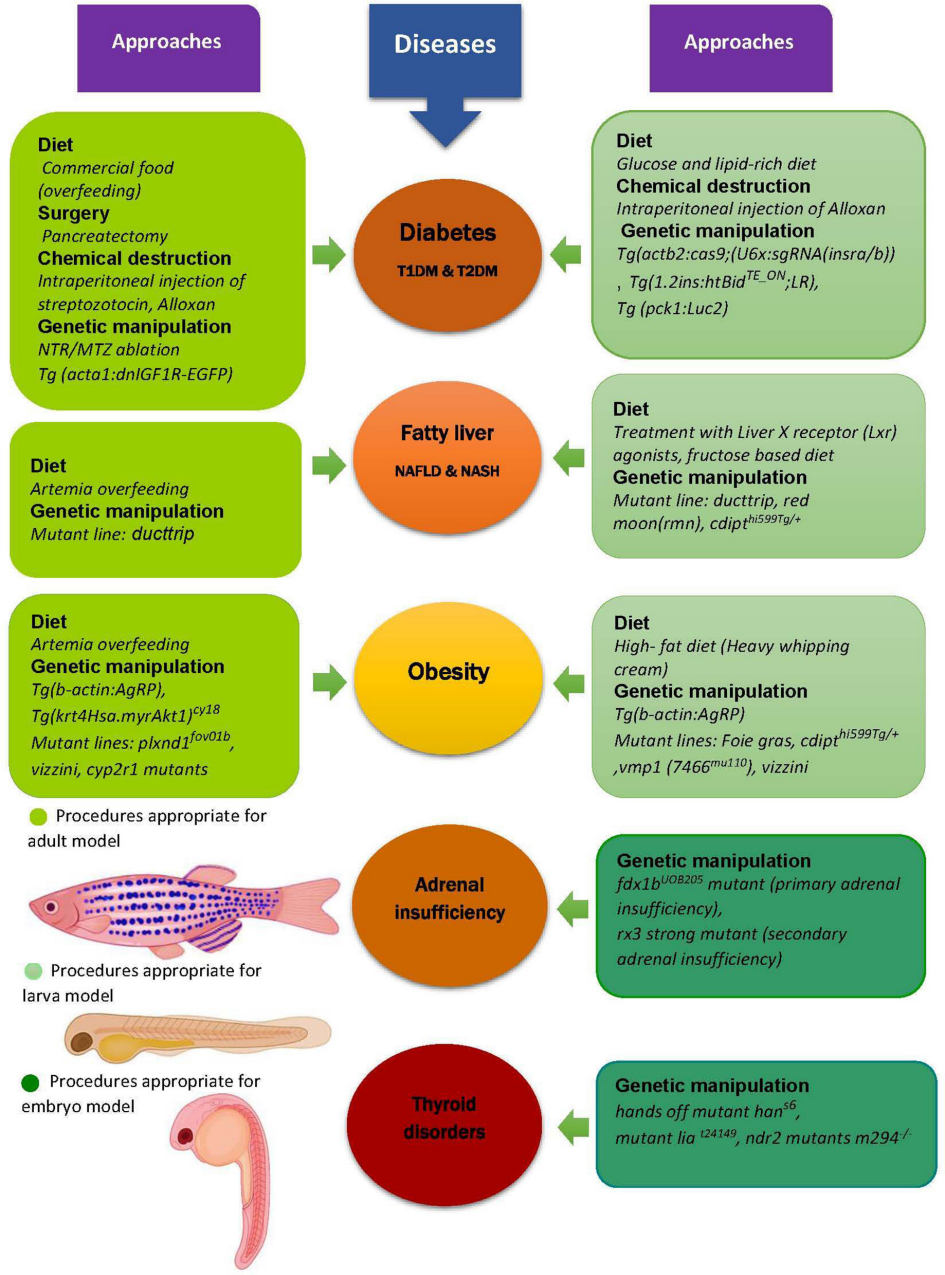
Zebrafish as a model for metabolic and endocrine diseases. Zebrafish is used as an appropriate model for preclinical study of different metabolic diseases like obesity, diabetes and fatty liver, and also some other endocrine disorders such as adrenal insufficiency and thyroid diseases. The models are generated by different approaches including diet, genetic manipulation, surgery, and chemical induced destruction of β cells in pancreas, some examples of which are shown in this figure. Additionally, it can be seen that there are different kinds of procedures used to model the diseases in each stage of zebrafish lifecycle. For instance, the diet of diabetic larva model is not similar to its adult model or various mutations (yet similar) result in fatty liver in larva and adult zebrafish. T1DM, Type1 diabetes mellitus; T2DM, Type2 diabetes mellitus; NAFLD, Non-alcoholic fatty liver disease; NASH, Nonalcoholic steatohepatitis; Tg, Transgenic line; IGF1R, Insulin-like growth factor1 receptor; EGFP, Enhanced green fluorescent protein; NTR, Nitroreductase; MTZ, Metronidazole; AgRP, Agouti-related protein; cyp2r1, Cytochrome p450 family2 subfamily R member1; plxnd1, Plexin D1; cdipt, Cytidine diphosphate-diacylglycerol-inositol 3-phosphatidyltransferase; pck1, Phosphoenolpyruvate-carboxykinase, Luc2, Luciferase gene; vmp1, Vacuole membrane protein1; il1b, Interleukin 1 beta; fdx1, Ferredoxin1; rx3, Retinal homeobox gene3; lia, Limabsent; ndr2, Nodal related gene (Cyclops) (9, 50, 62, 85, 86, 93–104).

The prevalence of obesity, type 2 diabetes mellitus (T2DM), NASH, and other endocrine and metabolic diseases which have increased over the past decades, causing a decrease in the quality of life of many people around the world. Since these disorders are complex conditions including genetic and environmental factors and they have no definite treatment, regenerative medicine (especially personalized regenerative medicine) has been investigated to find new therapies (3). In this respect, generating an appropriate animal model to investigate the unknown mechanisms and experimenting with drug and other therapeutic interventions is necessary. Among different animal models used over the past decades, zebrafish is considered as a well-established one for developing human genetics, biology, and diseases and it has been successfully used in this area, though it is still being validated (9, 13, 63, 78). Accordingly, some of the studied models of zebrafish DM, obesity, and NAFLD as symbols of metabolic and endocrine-related disorders are discussed. Moreover, in Figure 1, other examples of modeling strategies for these diseases in addition to examples of modeling two other endocrine dysfunctions, thyroid disorders, and adrenal insufficiency are illustrated.

### Zebrafish Model to Study Diabetes

There are different groups for studying DM in zebrafish models including: causing retinopathy by intubation in glucose-enriched water, inducing insulin resistance with different genetic mutations, β-cell ablation and other models that allow scientists to focus on any aspect of DM (50). Zebrafish pancreas, like mammals, consists of exocrine and endocrine parts, which are connected to the digestive tract. Also, it is capable of regenerating the pancreas, so, it has been used to study pathogenesis of both T1DM and T2DM (62). To apply β cell destruction for a T1DM model, three main methods are provided: surgery, chemical destruction, and genetic ablation (9, 93). Pancreatectomy is technically difficult, so it is not used very often in spite of being feasible in transgenic zebrafish which has green fluorescent protein (GFP) specific islet expression. Intraperitoneal injection of streptozotocin (STZ), a common way of chemical-induced diabetes in rodents, can effectively ablate β cells, although higher concentration is needed (9, 93). Alloxan is another chemical which can kill β cells in both adult zebrafish and its larvae (9). There are different genetic approaches, one is NTR/MTZ ablation system in which transgenic zebrafish with β cell expression of the bacterial NTR enzyme are exposed to the metronidazole (MTZ) which is the substrate of the enzyme and converts in to a cytotoxic compound inducing β cell apoptosis. Another approach is using a transgene in larvae that makes the insulin promoter to trigger the expression of a doxycycline/ecdysone-dependent transcription factor and the TetOR-based promoter to express the pro-apoptotic protein, Bid, to induce apoptosis. It should be noted that due to the substantial capacity of β cell regeneration in zebrafish, the β cell mass restores after removing the ablation mechanism (9). Insulin resistance and β cell dysfunction are two major characteristic of T2DM. Nutritional approach, along with genetic alteration were used to generate models, for instance, immersing zebrafish in glucose solution or over feeding it with a commercial food. For developing insulin resistant transgenic models, one way is to express a dominant-negative IGF-I receptor (IGF-IR) in skeletal muscle, causing increased fasting blood glucose [Transgenic line (Tg) (*acta1:dnIGF1R-EGFP*)]. Another example is using CRISPR/Cas9 to specifically knockdown the insulin receptor in liver Tg (actb2:cas9; (U6x:sgRNA(insra/b))), causing hyperglycemia after eating and hypoglycemia during fasting (9, 105). Recently, instead of the destruction of β cells and studying their regeneration, developing a model of islet inflammation—which is reported to be involved in the pathogenesis of diabetes- and understanding the protection mechanisms were introduced as another strategy. Since Interlukin-1 β (IL-1β) has a pivotal role in inflammation, Tg (ins:il1b) zebrafish were used to identify underlying processes leading to β cell dysfunction and its following consequences such as hyperglycemia. In addition, treatment with wedellactone, which is a natural product with anti-inflammatory properties, could reduce the β cell inflammation and infiltration of immune cells. Hence, this study opened a new window in investigating potential therapeutic approaches for β cell dysfunction and its associated disorders (94). Generally, zebrafish promotes the understanding of the underlying processes of DM pathogenesis for therapeutic approaches. In addition, due to its various potential benefits like regenerative capacity and its application in other areas of personalized medicine, it can be suggested as a personalized tool in this field (9, 63, 66).

### Zebrafish Model to Study Obesity

Obesity is the result of positive energy balance, when the energy intake is more than its expenditure it involves regulation among different organs such as brain, liver and adipose tissue. Since these organs and their interplay are quite similar in zebrafish, it has been used to model obesity (9, 62). Both zebrafish larva and its adult can be obese through overfeeding, which is a beneficial approach for reflecting the human pathology. The high fat diet increases adiposity in larva and over feeding with Artemia diet in adult fish showed hypertriglyceridemia, hepatosteatosis, and increased BMI (9, 13). Since genetic factor play an important role in this complex disorder, genetic models have been used via transgenic or mutant lines. Altering the pathways controlling body weight (like in mammals) is a way to generate models. The central melanocortin system (CMS), consists of proopiomelanocortin (POMC), peptides and agouti related peptide (AgRP), controls and regulates energy homeostasis, so, chromosomal translocation to suppress central melanocortin receptors is one of these models. Transgenic zebrafish [Tg (b-actin:AgRP)] exhibit its increased weight, visceral adipose accumulation, and total triglyceride content as the result of overexpressing AgRP, an endogenous antagonist of melanocortin (9, 95). In addition, mutant lines like *Foie gras* [essential for endoplasmic reticulum (ER) to Golgi vascular transport] and cdipt^hi599Tg/+^ (cytidine diphosphate-diacylglycerol-inositol 3-phosphatidyltransferase which causes lipid accumulation in hepatocytes) often cause fatty liver at larval stages, mostly because of ER stress. Moreover, some mutants change the adipose tissue. For instance, the somatic growth decreases in the zebrafish *vizzini* mutant while subcutaneous and visceral adipose tissues are increased. This is because of a mutation in growth hormone 1 gene (gh1). Another mutation in cytochrome p450 family2 subfamily R member1 (cyp2r1) gene which makes the deficiency of 1α,25(OH)_2_D3 (the active form of vitamin D), increases adiposity and causes growth retardation (9). Overall, genetic manipulation in zebrafish is considered to be a beneficial strategy for characterizations of genetic basis and mechanisms responsible for storage and mobilization of adipose tissue which can help target specific molecular pathways in order to treat or prevent obesity possibly in individuals in personalized regenerative medicine (9, 13). Some of the other genetic manipulations for developing obesity model are exhibited in Figure 1. Besides, there is a correlation between intestinal microbiota and obesity and its related disorders like NAFLD (13, 91). Also, gut microbiota as a unique feature in humans has gained attention in the field of personalized medicine (90). Accordingly, the similarity of composition of the gut microbiota in zebrafish has made germ-free zebrafish a useful and attractive model for research on the intestinal microbiota (13). Moreover, the effect of dietary lipid content on the gut microbiota of zebrafish revealed that HFD has a negative effect on gut microbiota and supplementation of the probiotic *Lactobacillus rhamnosus*, which influences appetite control and lipid metabolism in zebrafish, was able to change the transcription of genes related to cholesterol and triglyceride metabolism and controlling appetite. It could decrease the transcription of these genes and reduce weight gain in HFD and medium fat diet (MFD) fed group (13, 92, 106). These data exhibit the role of gut microbiota in obesity and suggest new alternatives for treating obesity and its related disorders in metabolism, which can be applied in a personalized approach (13, 90). Taken together, zebrafish as an emerging obesity model is a convenient study tool to identify the effects of dietary supplements on weight and body fat and investigate the mechanisms and pathologic basis of obesity (9). Although there are some limitations such as lack of a standardized diet, these animals can be useful for testing novel therapeutic approaches, likely in personalized regenerative medicine concerning underlying mechanisms like modulating the gut microbiota (13, 90).

### Zebrafish Model to Study Fatty Liver Disease

NAFLD is the result of excessive accumulation of lipids in the liver, which can lead to NASH, cirrhosis and cancer (50). It is proved that it has association with obesity and insulin resistant but there are still unknown mechanisms for its progression (62). For further investigations, there are some zebrafish mutants being used, for example the *ducttrip* line, with the mutation in s-adenosylhomocystein hydrolase (AHCY) which is an essential metabolic enzyme involved in methylation reactions, shows degeneration of the liver and hepatic steatosis. Patients with AHCY deficiency- a rare genetic disorder-not only have liver dysfunction but also some impairments in brain function, therefore, this model can be helpful to identify new therapeutic options for the patients (62, 78, 85). Also, some kinds of diets such as ketogenic and fructose based ones can trigger steatosis (50).Additionally, various transgenic tools can be used to study hepatic cell types and even adipocytes for better understanding of NAFLD pathogenesis (50, 85). Consequently, the zebrafish acts as a powerful model and system to explore the pathways leading to NAFLD and uncover key parts (85). It should be mentioned that not all of these approaches are appropriate for three main stages of zebrafish lifecycle (embryo, larva, and adult), although there might be some similarities. Therefore, Figure 1 shows some of different approaches experimented to model the metabolic diseases which were mentioned in the text, and some other endocrine disorders.

### Limitations in Zebrafish Models of Metabolic Diseases

There are more obstacles in using zebrafish as a metabolic disease model than in other disorders like cardiovascular studies. Exclusive human factors affecting metabolism such as lifestyle, behavioral, and socioeconomic factors cannot be replicated in zebrafish. Zebrafish diet is also very difficult to track individually and since their growth is dependent on many uncontrollable factors including temperature, body fat, genetics, etc. it is not an accurate factor for measuring intake. Due to limitations in repeating blood sampling, metabolism assays like hormone testing and insulin tolerance tests are all much harder to conduct on zebrafish (50). Eventually, there are some challenges in performing specific procedures such as intraperitoneal injections in zebrafish that are easily achievable in other animal models like mice (62).

## Conclusion

Cell therapy and regenerative medicine are the major causes of remarkable revolution in different fields of medicine in recent decades. The purpose of these advanced therapies to cure disease and regenerate impaired tissues and organs rather than just treat and repair them (107, 108). Hence, the development of these novel technologies could lead to astonishing advances in biomedicine. Accordingly, a worldwide effort should be made in various aspects to attain this goal. In this context, standard preclinical researches, as a fundamental step, should be implemented to properly investigate different procedures in order to reach a more applicable protocol translation to clinical settings. For this purpose, more compatible animal models should be chosen depending on the particular aim of the study (93, 105). In regeneration studies, the selected model should possess the ability of regeneration in a distinct part of the body according to the study goal. For instance, in the case of type 2 diabetes, pancreatic dysfunction occurs due to a decrease in the number of islets and consequently β-cells. Since the cells responsible for insulin secretion undergone a decrease, the need for exogenous insulin rises. However, insulin injection is not working for a long time and results in some troubles for patients. Therefore, a novel therapy to restore pancreatic function without the need for exogenous insulin seems an ideal escape route. Unfortunately, the human does not have regeneration ability in many organs and tissues. Therefore, in order to trigger regeneration activity in human tissues, an exogenous stimulus is needed. Another way is the application of exogenous cells or tissues that need to be carefully tested because of their unexpected results (109, 110). Hence, to put these peculiar mechanisms away from the human ethical troubles, regeneration prone animal models should be identified. Using animal models, we can provide safety and efficacy evidence and a cost-effective translational bridge will be provided. Obviously, there is no perfect animal model with the ability to completely mimic all characteristics of the human body and disease conditions. Here, given its inevitable limitations, zebrafish with the potency for pancreas regeneration represents an efficient regeneration in β-cells that makes a suitable model for targeting signaling pathways to study the regeneration of β-cells through different stimulations. Additionally, the genomic similarity of zebrafish to humans will be a precious tool for genetic manipulation and developing transgenic models for ascertaining health improvement procedures through personalized medicine studies (9, 93). Altogether, using a humanized zebrafish model, it would be possible to investigate existing novel therapies such as cell therapy and regenerative medicine for identifying the optimal treatment for each individual. Ultimately, zebrafish will be a powerful model for implementing personalized cell-based treatments, since the advantages of using zebrafish as a humanized disease model to investigate disease mechanisms and drug efficacy can help us overcome the drawbacks of using mice models, the interest in the former is growing rapidly (69). So far, cancer clinical trials are the field in which zebrafish is most prevalently used in personalized medicine (USANI) (11), but the effort is being made to use zebrafish in endocrine diseases. The regenerative potential, the genetic similarity with humans, and the opportunity to study mechanisms in a more comprehensive way has led us to believe that zebrafish can be a suitable model to move us further in the field of regenerative personalized medicine.

## Future Perspectives

Thanks to zebrafish models, in recent years, investigations in the field of human diseases faced remarkable progressions that revealed the importance of its employment in different studies. Recent investigations have been implemented in zebrafish in endocrine-related disorders using omics technologies. For instance, biomarker alterations in different endocrine disruptions have been detected and screened using proteomics in this model. In fact, due to specific characteristics of zebrafish in providing the possibility of rapid screening with the ability to distinguish molecular changes based on sex and organ specification, it can be considered as a suitable choice in this regard (111). Moreover, since zebrafish can be utilized for high-throughput non-biased chemical screening, there is a growing rate in its employing for *in vivo* drug discovery researches (112). Interestingly, its notable similarity to humans and providing the accessibility to a wide set of genetic tools for gene mutation, in addition to its rapid development and limited ethical issues introduces zebrafish as a potential choice for individualized drug therapy studies (113). Nowadays, the utilization of zebrafish for precision medicine research, especially in endocrine diseases, is rapidly emerging. Up to now, several zebrafish cancer studies have been conducted for developing different humanized models (using xenografts), target validation, investigating the safety, toxicology, and effectiveness of drugs that it is growing significantly in other fields (114, 115). The outlook of using zebrafish in personalized regenerative medicine seems promising due to the same advantages which zebrafish has in cancer studies. Furthermore, advances in visualization methods, gene targeting, and chemical screening in zebrafish models can improve future perspectives and findings.

## Author Contributions

BA contributed substantially to the conception and design of the study. AT-B, NF, and SA drafted critical revision of the article. MP conducted search strategy and data collection. SA-M provided final approval of the version to publish. MG and PG revised the article critically for important intellectual content. BL give final approval of the version to be submitted and any revised version. All authors contributed to the article and approved the submitted version.

## Conflict of Interest

The authors declare that the research was conducted in the absence of any commercial or financial relationships that could be construed as a potential conflict of interest.

## Abbreviations

ADHD: Attention deficit hyperactivity disorder
AgRP: Agouti related peptide
AHCY: S-Adenosylhomocystein hydrolase
BECs: Biliary epithelial cells
BMP: Bone morphogenetic protein
CNS: Central nervous system
*C. elegans*: *Caenorhabditis elegans*
cyp2r1: Cytochrome p450 family2 subfamily R member1
cdipt: Cytidine diphosphate-diacylglycerol-inositol 3-phosphatidyltransferase
CMS: Central melonocortin system
CRISPR: Clustered regularly interspaced short palindromic repeats
DM: Diabetes mellitus
T1DM: Type1 diabetes mellitus
T2DM: Type2 diabetes mellitus
fdx1: Ferredoxin1
ESCs: Embryonic stem cell
EKG: Electrocardiogram
EGFP: Enhanced green fluorescent protein
FGF: Fibroblast growth factor
GABA: Gamma-amino butyric acid
gh1: Growth hormone 1 gene
GFP: Green fluorescent protein
HPSCs: Human pluripotent stem cells
HGF: Hepatocyte growth factor
IGF: Insulin-like growth factor
IL-1β: Interlukine-1β
Luc2: Luciferase gene
lia: Limabsent
Lxr: Liver x receptor
MAP: Mitogen-activated protein
MI: Myocardial infarction
MSCs: Mesenchymal stem cells
MTZ: Metronidazole
3-MPA: 3-Mercaptopropionic acid
NASH: Non-alcoholic steatohepatosis
NAFLD: Non-alcoholic fatty liver disease
NTR: Nitroreductase
ndr2: Nodal related gene (Cyclops)
PGE2: Prostaglandin E2
POMC: Proopiomelanocortin
plxnd1: Plexin D1
PCK1: Phosphoenolpyruvate-carboxykinase1
IGF-IR: IGF-I receptor
iPSCs: Induced pluripotent stem cells
rx3: Retinal homeobox gene3
rmn: Red moon
STZ: Streptozotocin
Tg: Transgenic line
TALEN: Transcription activator-like effector nuclease
VEGF: Vascular endothelial growth factor
vmp1: Vacuole membrane protein1
uhrf1: Ubiquitin like with PHD and finger domain1
WNT: Wingless-related integration site
EDCs: Endocrine-disrupting components
LPS: Lipopolysaccharides.
